# A functional single nucleotide polymorphism of SET8 is prognostic for breast cancer

**DOI:** 10.18632/oncotarget.9099

**Published:** 2016-04-29

**Authors:** Ben Liu, Xining Zhang, Fengju Song, Qun Liu, Hongji Dai, Hong Zheng, Ping Cui, Lina Zhang, Wei Zhang, Kexin Chen

**Affiliations:** ^1^ Department of Epidemiology and Biostatistics, Key Laboratory of Breast Cancer Prevention and Therapy, Ministry of Education, Key Laboratory of Cancer Prevention and Therapy, Tianjin, National Clinical Research Center for Cancer, Tianjin Medical University Cancer Institute and Hospital, Tianjin 300060, China; ^2^ Departments of Neurosurgery, Key Laboratory of Breast Cancer Prevention and Therapy, Ministry of Education, Key Laboratory of Cancer Prevention and Therapy, Tianjin, National Clinical Research Center for Cancer, Tianjin Medical University Cancer Institute and Hospital, Tianjin 300060, China; ^3^ Department of Pathology, The University of Texas MD Anderson Cancer Center, Houston, TX 77030, USA; ^4^ Department of Cancer Biology, Comprehensive Cancer Center of Wake Forest Baptist Medical Center, Winston-Salem, NC 27157, USA

**Keywords:** SET8, single nucleotide polymorphisms (SNP), rs16917496, prognosis, breast cancer

## Abstract

A single-nucleotide polymorphism (SNP) locus rs16917496 (T > C) within the 3′-untranslated region (3′-UTR) of SET8 was associated with susceptibility in several malignancies including breast cancer. To further elucidate the prognostic relevance of this SNP in breast cancer, we conducted a clinical study as well as SET8 expression analysis in a cohort of 1,190 breast cancer patients. We demonstrated the expression levels of SET8 in TT genotype were higher than in CC genotypes, and high levels of SET8 were associated with poor survival. SET8 expression was significantly higher in breast tumor tissue than in paired adjacent normal tissue. In addition, survival analysis in 315 patients showed SNP rs16917496 was an independent prognostic factor of breast cancer outcome with TT genotype associated with poor survival compared with CC/CT genotypes. Thus, this SNP may serve as a genetic prognostic factor and a treatment target for breast cancer. Future studies are warranted.

## INTRODUCTION

Breast cancer is the most common malignancy in women in many countries, including China [1, 2]. In spite of the generally good prognosis of breast cancer patients, wide variation in survival indicates that genetic factors may affect the outcomes of breast cancer [3, 4]. Thus, exploring potential genetic biomarkers could benefit to a more appropriate prediction and effective therapeutic strategy of breast cancer prevention and treatment.

In 2008, the first epidemiological study demonstrated that single-nucleotide polymorphisms (SNPs) at microRNA (miRNA) -binding sites within the 3′–untranslated (3′-UTR) region were associated with cancer risk [5], prompting us to propose the polymorphisms in microRNA targets were a gold mine for molecular epidemiology [6]. Afterward numerous investigations have revealed the SNPs within miRNA-binding region may contribute to cancer risk and outcomes by altering miRNA-mRNA binding affinities and miRNA-targeted gene expression [7, 8]. For example, He *et al.* found that rs8752 located with *miR-485-5p*-binding site of *HPGD* gene was related to the risk of breast cancer [9]. Zhang *et al.* found that *miR-367*-binding site rs1044129 in *RYR3* gene was associated with poor survival of patients with breast cancer [10].

SET8 (also known as SETD8, PR-SET7, KMT5A), located on chromosome 12q24.31, is a specific histone H4 lysine 20 methyltransferase (H4K20me1) [11]. Prior studies indicated SET8 may exert various functions in a series of biological processes, including maintaining genome integrity [12], controlling cell-cycle progression and development [13], regulating gene transcription [14], and mediating DNA repair and damage through its histone monomethylating activity [15]. Moreover, SET8 was also found to bind and methylate nonhistone proteins such as p53, TWIST, Wnt-activated genes, PCNA, ERα and AR [16–21]. All of the above discoveries suggested that SET8 may have a link with carcinogenesis and cancer progression.

Based on a large case control cohort, we first demonstrated that SNP rs16917496-T/C located in the 3′UTR of the SET8 mRNA was associated with the risk of early onset of breast cancer, and this SNP region was predicted as a potential binding site of *miR-502* [22]. This SNP was subsequently shown by others to be a susceptibility factor for a number of cancers, including non-small cell lung cancer [23], epithelial ovarian cancer [24], childhood acute lymphoblastic leukemia [25], and cervical cancer [26]. This broad spectrum of association suggests that this SNP is a robust genetic regulatory factor fundamental to cells. However, the role of the SET8 3′-UTR SNP in breast cancer prognosis has remained unclear and has not been reported, which is the main motivation of this investigation.

## RESULTS

### Clinical characteristics of breast cancer patients

A total of 1,190 pathologically confirmed breast cancer patients were enrolled in the study. The demographic and clinical characteristics of patients were summarized in Table 1. The median age at diagnosis was 54 years (range, 29–89 years). The median follow-up time of the 315 breast cancer patients who had complete follow-up information from this cohort was 82 months (range, 78–115 months). During the follow-up period, 26 patients died from breast cancer, and 14 patients were loss follow-up. Of these 315 patients, the expression of SET8 mRNA in 30 pairs of tumor and adjacent normal sample was analyzed.

**Table 1:** Association of SNP in SET8 3′-UTR and clinicopathological features of 1190 breast cancer patients

Variable	Number	CC + CT (%)	TT (%)	*P* value
**Age at diagnosis(years)**				
** < 50**	582	335(57.6)	247(42.4)	0.138
** ≥ 50**	608	324(53.3)	284(46.7)	
**Tumor size (cm)**				
** < 2**	372	209(56.2)	163(43.8)	0.647
** ≥ 2**	538	294(54.6)	244(45.4)	
**TNM stage**				
** I + II**	511	288(56.4)	223(43.6)	0.700
** III**	85	46(54.1)	39(45.9)	
**Lymph node metastasis^a^**				
** No**	682	386(56.6)	296(43.4)	0.302
** Yes**	480	257(53.5)	223(46.5)	
**Histological grade^a^**				
** I + II**	665	368(55.3)	297(44.7)	0.295
** III**	131	79(60.3)	52(39.7)	
**Family history of breast cancer^a^**				
** No**	779	423(54.3)	356(45.7)	0.097
** Yes**	323	193(59.8)	130(40.2)	
**Menopause^a^**				
** No**	525	292(57.0)	226(43.0)	0.426
** Yes**	570	311(54.6)	259(45.4)	
**ER^a^**				
** Negative**	495	292(59.0)	203(41.0)	0.011^*^
** Positive**	606	311(51.3)	295(48.7)	
**PR^a^**				
** Negative**	446	252(56.5)	194(43.5)	0.352
** Positive**	656	352(53.7)	304(46.3)	
**HER2^a^**				
** Negative**	697	380(54.5)	317(45.5)	0.420
** Positive**	334	191(57.2)	143(42.8)	
**Molecular subtype^a^**				
** Luminal type**	494	259(52.4)	235(47.6)	0.118
** HER-2 overexpression**	137	80(58.4)	57(41.6)	
** Basal-like**	171	104(60.8)	67(39.2)	
**PCNA^a^**				
** +**	130	71(54.6)	59(45.4)	0.767
** ++** ** +++**	746 63	414(55.5) 32(50.8)	332(44.5) 31(49.2)	
**P53^a^**				
** Negative**	626	335(53.5)	291(46.5)	0.293
** Positive**	392	223(56.9)	169(43.1)	

a Total case number was less than 1190 owing to missing data;

* Indicate statistically significant (*P* < 0.05).

### The association of SET8 expression with T allele and poor outcome

**Figure 1:**
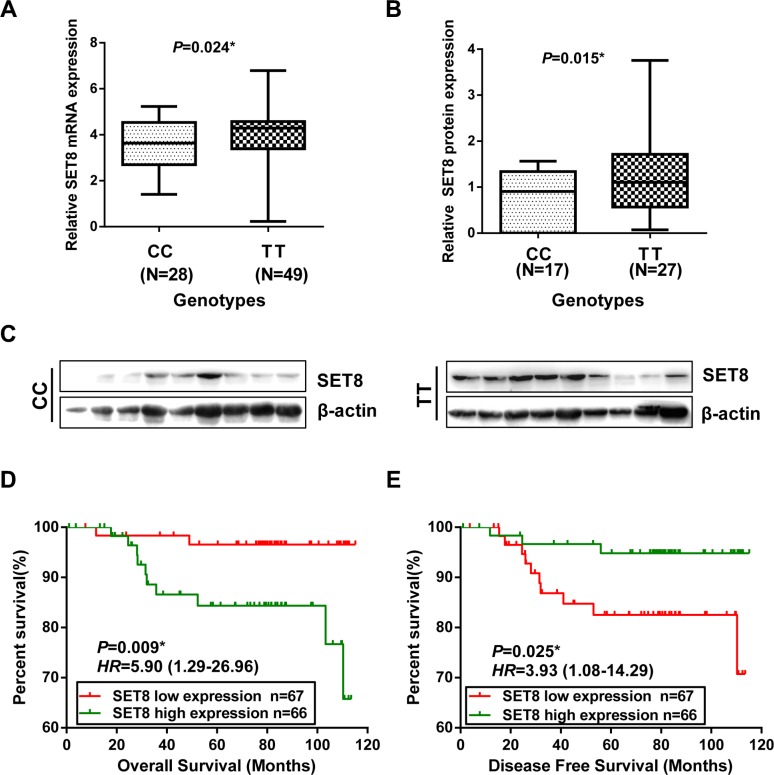
Functional relevance of SET8 3′-UTR SNP on SET8 expression and the association of SET8 expression with the prognosis of breast cancer patients (**A**) The relative expression level of SET8 mRNA in breast cancer tumor tissues measured by taqman qRT-PCR in 77 breast cancer patients. Housekeeping gene GADPH was used as the reference. (**B**) The graph represents the relative quantitation of protein expression of SET8 in tumor tissues by Western blot in 44 breast cancer cases. (**C**) The image shows the representative Western blot result of SET8 expression between two genotypes from panel B. (**D**) Kaplan–Meier curves of overall survival of breast cancer patients with high or low SET8 expression level. (**E**) Kaplan–Meier curves of disease free survival of breast cancer patients with high or low SET8 expression level. *P* values are from the log-rank test. HR with 95% CI was from Univariate analysis of OS and DFS.

In order to evaluate the biological relevance of the rs16917496 polymorphisms, we examined SET8 relative expression through semi-quantitative RT-PCR (qRT- PCR) in 77 breast cancer patients with different SET8 genotypes. The results showed that breast tumors had higher expression of SET8 mRNA in TT genotype than CC genotype (*P* = 0.024) (Figure 1A). In addition, we also measured the protein expression of SET8 in tumor tissues by Western blot in 44 patients, the results indicated that the SET8 protein in TT genotype was higher than CC genotype (*P* = 0.015) (Figure 1B and 1C).

To further elucidate the correlation of SET8 expression with overall survival (OS) and disease-free survival (DFS), we performed Kaplan-Meier analysis by stratifying patients according to SET8 median expression. Kaplan-Meier survival curves suggested that patients with high expression of SET8 had poor OS and DFS compared with the SET8 low expression group (*P* = 0.009 and *P* = 0.029, respectively) (Figure 1D and 1E). Analysis by univariate Cox proportional hazard model also showed similar results both in OS (HR = 5.90; 95% CI: 1.29– 26.96) and DFS analysis (HR = 5.90; 95% CI: 1.08– 14.29) (Figure 1D and 1E).

### The mRNA expression of SET8 in breast cancer tissues

**Figure 2:**
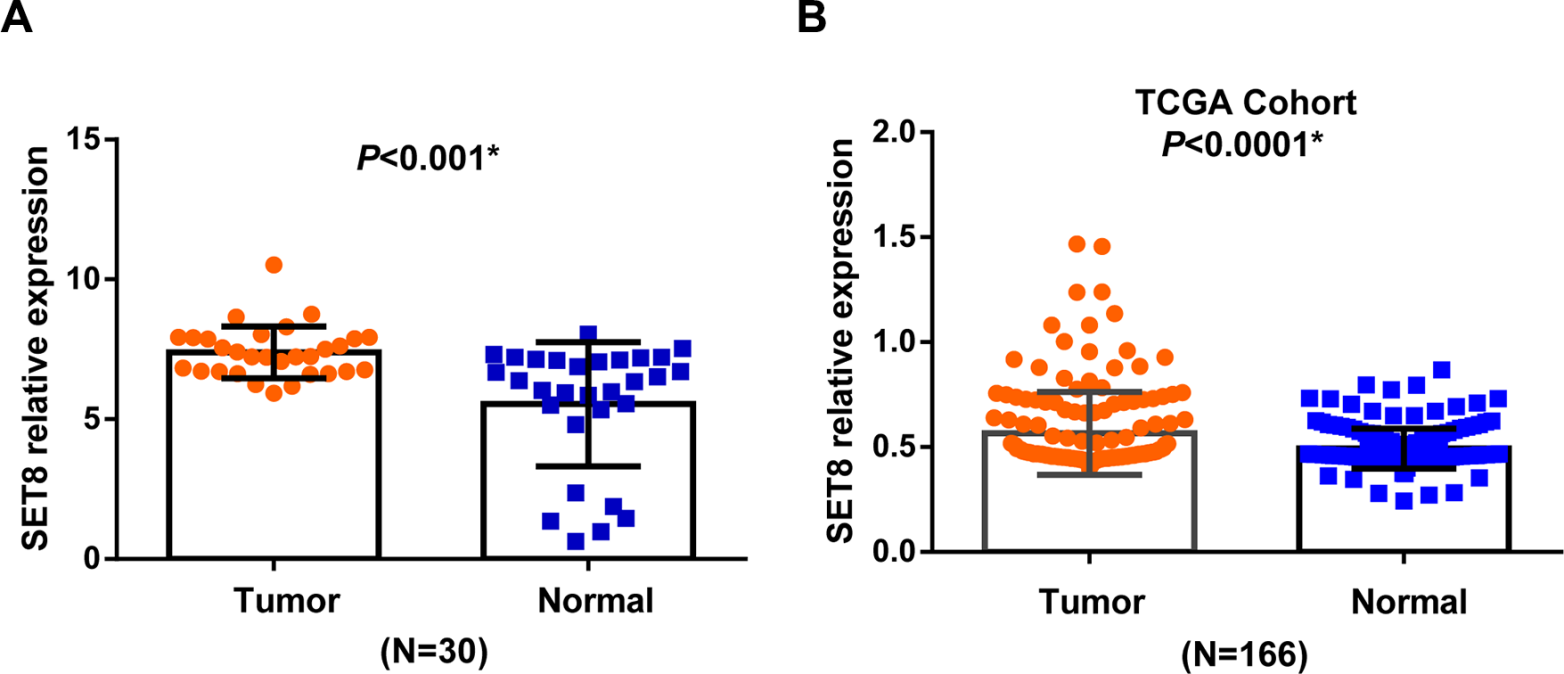
The mRNA expression of SET8 in breast cancer tissues and paired normal tissues (**A**) The relative expression SET8 in 30 pairs of breast cancer tissues and paired normal tissues by qRT-PCR. (**B**) The validation results in 166 pairs of breast cancer tissues and paired normal tissues from TCGA database.

We subsequently evaluated the relative expression of SET8 in 30 pairs of breast cancer tissues and paired normal tissues. The result suggested that the relative expression of SET8 was up-regulated in breast cancer tissues than in paired normal tissues (*P* < 0.001) (Figure 2A). This result was also validated in the TCGA cohort of 166 pairs of breast cancer tissues and normal tissues (*P* = 0.03) (Figure 2B).

### The protein expression of SET8 in breast cancer tissues

**Figure 3:**
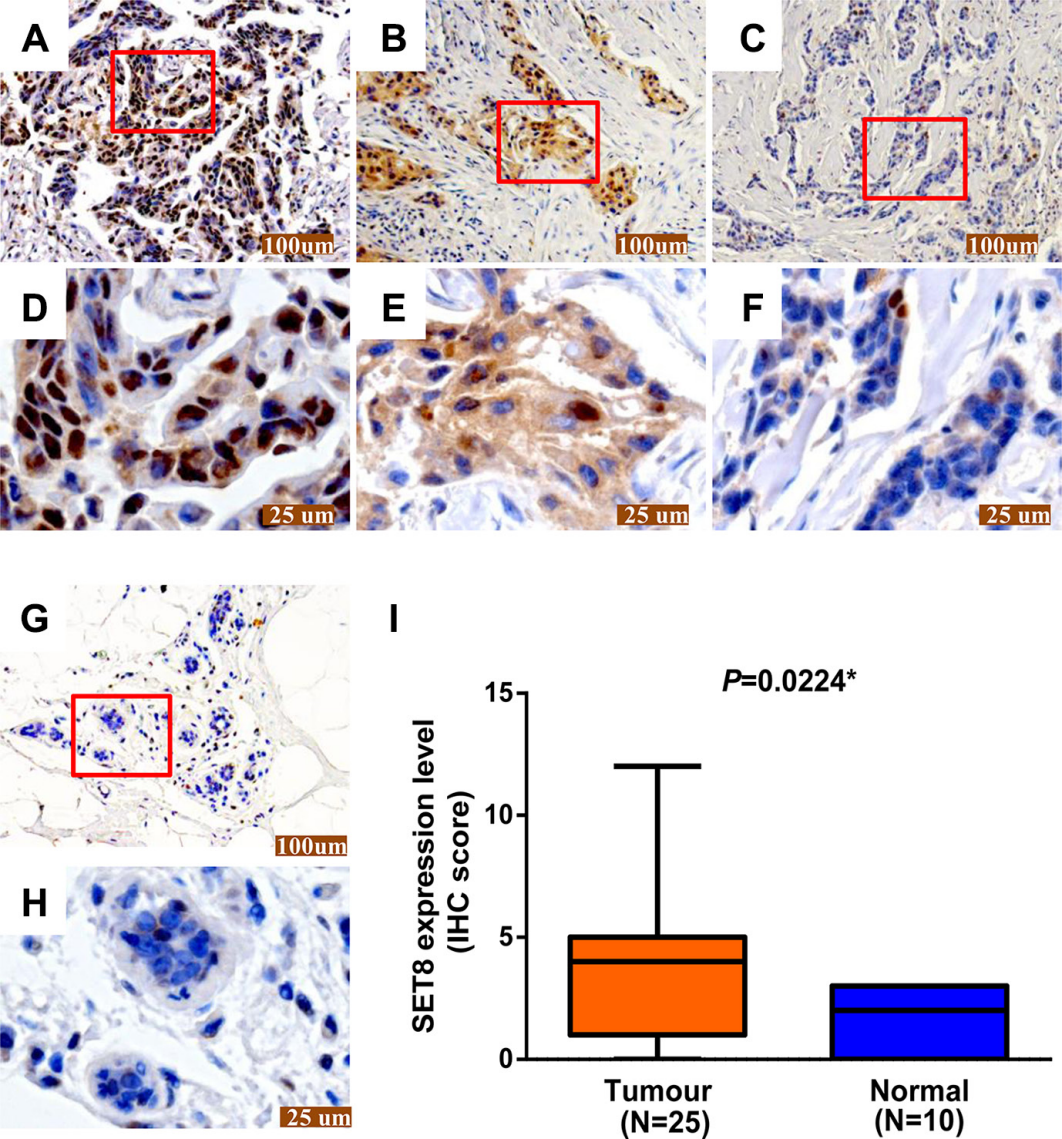
The SET8 protein expression in breast cancer tissues and adjacent normal tissues (**A–C**) Representative images of different degrees of staining was detected by immunohistochemical staining in breast cancer tissue sections: (A) strong, (B) moderate, (C) weak. (**D–F**) Higher magnification images of A–C, respectively. (**G**) Representative images of SET8 protein expression in breast adjacent normal tissues were detected by immunohistochemical staining. (**H**): Higher magnification images of G. Scale bar represents 100 μm in 100× magnification (A-C and G) and 25 μm in 400× magnification (D-F and H), respectively. (**I**) Semi-quantitative levels of immunohistochemical staining (IHC score) in samples of breast cancer tissues and adjacent normal tissues. *P < 0.05

In addition, expression of SET8 protein was also examined in 25 breast cancer tissues (Figure 3A–3F) and 10 adjacent non-cancerous tissues (Figure 3G and 3H) by immunohistochemical staining. SET8 protein was strongly expressed in the nucleus in breast cancer tissue. The results showed that the protein levels of SET8 were significantly higher in breast cancer tissues compared with the adjacent non-cancerous tissues (*P* = 0.022) (Figure 3I).

### Association of T allele with poor outcome

**Figure 4:**
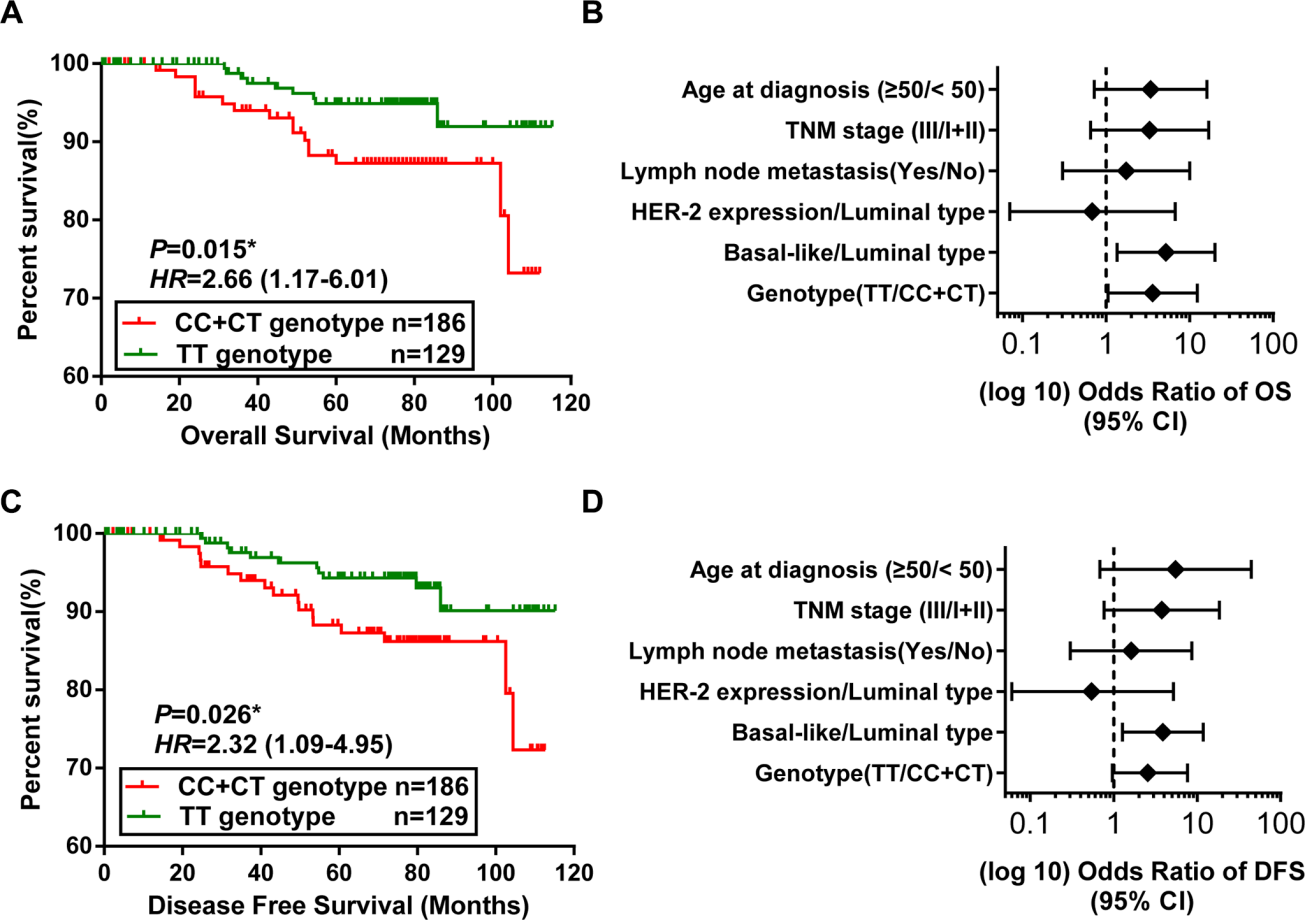
Association between the SNP in SET8 3′-UTR and breast cancer survival (**A**, **C**) Kaplan-Meier analysis of overall survival (OS) (A) and disease free survival (DFS) (C) of patients with the CC + CT phenotypes vs. the TT genotype in 315 cases. Red line represents CC and CT genotypes, and the green line represents TT genotypes. *P* values are from the log-rank test. HR with 95% CI was from Univariate analysis of OS and DFS. (**B**, **D**) Forest plot of the association of breast cancer prognosis factors with OS (B) and DFS (D) of 315 breast cancer patients. The results were from Cox regression analysis. The confounding factors include age, TMN stage, lymph node metastasis, and molecular subtype.

The association between the rs16917496 genotypes and the clinical and pathological features of 1,190 breast cancer patients was shown in Table 1. We found that the rs16917496 genotypes were associated with ER status (TT vs. CC + CT, *P* = 0.011), and the expression of p53 was significantly lower in CC genotype than in CT and TT genotypes (*P* = 0.047) (Supplementary Figure 1). To determine whether the rs16917496 genotype was associated with the outcome of breast cancer patients, we analyzed the association of rs16917496 genotypes with overall survival (OS) and disease-free survival (DFS) in 315 breast cancer patients who had sufficient follow- up data. Kaplan-Meier survival curves suggested that compared with the TT genotype, the genotypes with C allele was significantly associated with longer OS and DFS of breast cancer patients (Figure 4A and 4C). After adjustment for potential confounding factors including age, TMN stage, lymph node metastasis, and molecular subtype, the multivariate Cox regression analysis was performed to evaluate the factors for breast cancer prognosis. Among all prognosis factors, TT genotype of rs16917496 was found to be significantly associated with increased risk of cancer-related death. The relative risk (RR) was 3.61, with 95% confidence interval (CI) ranging from 1.05 to 12.35. As expected, age at diagnosis, TNM stage, Lymph node metastasis, p53 status and molecular subtype was also significant predictors of outcome (Figure 4B). Multivariate Cox regression analysis showed that rs16917496 genotype (TT vs. CC + CT) was marginally associated with DFS of breast cancer (HR = 2.56, 95% CI: 0.96–7.65) (Figure 4D).

## DISCUSSION

In this study, we uncovered that TT genotype of rs16917496 on SET8 3′-UTR region was significantly associated with poor outcome of breast cancer in a Chinese population, and had allele-specific increased on SET8 expression. More importantly, these results remained significant after adjustment for other potential predictors of patient outcome in this patient cohort study. To the best of our knowledge, this is the first epidemiological study investigating the association of this SNP with breast cancer survival.

A growing number of researchers suggest that polymorphisms within miRNA-binding sites in 3′-UTR of genes contribute to carcinogenesis and progress by affecting miRNA-regulated gene expression [27]. In addition to the risk of cancer, the SNP rs16917496 has been related to the clinical outcome of several cancer types. For example, Ding et al. found the small cell lung cancer patients with SET8 CC + CT genotypes have good prognosis [28]. In another study, Guo et al. has demonstrated liver cancer patients with SET8 CC genotype postoperatively live longer than those without [29]. In non-Hodgkins lymphoma (NHL), Diao et al. found patients carrying SET8 CC genotype have significantly longer survival time than the patients with SET8 CT or TT genotype [30]. In line with these studies, our results discovered that the functional SNP associated with breast cancer survival via influencing SET8 expression. One rational explanation was *miR-502* bound more tightly to the C allele of rs16917496 than to the T allele. To test this hypothesis, We used four miRNA target predicting databases (miRWalk, TargetScan, miRMap and miRanda) to identify the candidate targets of miR-502. We found that the four programs all predicted SET8 as candidate targets of miR-502 and SNP16917496 located within the miR-502-binding site (Supplementary Figure 2A and 2B). In addition, we computationally determined the different minimum free energy (MFE) for both the SET8 genotype using an online thermodynamic software RNAHYBRID. The MFE of genotype C [mfe = −26.8 kcal/mol] was smaller than that of the T genotype [mfe = −25.1 kcal/mol], which suggested that miR-502 has a higher binding affinity for the C genotype (Supplementary Figure 2C and 2D). We went further to examine SET8 protein level in breast cancer patients with different rs16917496 genotypes. A significant increase was observed in both SET8 mRNA and protein levels in TT genotype relative to the CC genotype. One immunohistochemical study on 192 NSCLC tissue aligned with our results in which SET8 protein expression level of CC genotype found to be low [31]. By searching the Genotype-Tissue Expression (GTEx) portal database (http://www.gtexportal.org/home/), we performed an expression quantitative trait loci (eQTL) analysis to verify the association between the genotype and SET8 gene expression level in a cohort of 183 normal breast tissues. The result showed the SET8 expression significantly reduce in CC genotype compare to TT genotype (*P* = 0.021), which is consistent with our finding. The above evidence supports the hypothesis that functional 3′-UTR SNP harbor in miRNA target sites may regulate the miRNA-target gene expression through affecting the binding affinity, which may in turn affect the inhibitory regulation ability of miRNA on mRNA's translation to protein. In addition, these empirical findings consistently corroborate our result that the T allele of rs16917496 alters SET8 functions and therefore is associated with breast cancer outcome.

In our results, the data also suggested the expression of SET8 has a significant association with prognosis of breast cancer. SET8 is known as the sole methyltransferase for H4K20me1, which usually associates with the higher-order chromatin structure and the variability of actively transcribed genes in cancerous conditions as the epigenetic mark [32, 33]. Accumulative clinical data showed that the abnormal expression of histone methyltransferase is correlated to cancer occurrence as well as prognosis of various cancers. For example, the expression level of histone lysine methyltransferase EZH2 which mainly catalyze H3K27me3 is higher in the breast cancer, prostate cancer and bladder cancer, and has been used as a prognostic marker in breast cancer and metastatic prostate cancer [34, 35]. Elevated expression of Suv39h1, another histone methyltransferase which can catalyze H3K9me2, has been found to associate with higher incidence of hepatocellular carcinoma recurrence [36]. As recently more and more research groups devote to explore molecule targeting inhibitors of histone methyltransferase, such as EZH2- H3K27 inhibitor EPZ – 6438 [37] and DOT1L- H3K79 methylation inhibitors EPZ– 5676 [38], it has become a promising target for anti-tumor therapy [39, 40]. In summary, the result from our current study as well as that in other researcher's work revealed that SET8 closely associated with the occurrence of a wide variety of tumor development and prognosis, and therefore, is believed to become potential new targets for the treatment of tumor.

While interpreting these results and presenting the contributions of this study, several limitations should be considered and discussed as well. First, based on the accumulated evidence, we infer that SET8 has a key role in breast cancer progression by inducing cancer cell proliferation and migration. This implication needs further in-depth functional experimental research to investigate and help understand the complicated mechanism *in vitro* and *in vivo*. Second, because the sample size of survival and SET8 expression analysis was relatively modest or small, replication studies in a larger population are needed in order to validate our results. Third, our results suggested the SET8 rs16917496 genotype was associated with ER and p53 expression, which cannot be easily explained for regulation of protein methylation by SET8 due to the fact that it will not change the transcriptional level of these genes. Thus, the role of this SNP as a modifier needs further examination and clarification through laboratory-based functional studies.

In summary, we confirmed that the rs16917496 polymorphism can predict breast cancer patients' survival in Chinese population. The genetic variation rs16917496 with SET8 3′-UTR could modify the breast cancer outcome by regulating the expression of SET8. These findings present new evidence that the genetic variant might be a molecular switcher to fine tune the miRNA-mRNA binding and related gene function through a genetic regulatory circuit. Therefore, SET8 may be used as a new treatment target and prognosis biomarker for breast cancer.

## MATERIALS AND METHODS

### Ethics statement

The study had been approved by the Ethical Committee of Tianjin Medical University Cancer Institute and Hospital. The informed consent form was signed by each participant.

### Study subjects

A total of 1,190 newly diagnosed primary breast cancer cases was recruited from Tianjin Medical University Cancer Institute and Hospital between 2001 and 2008. Demographic features and clinical information were separately collected from structured questionnaire and medical records. The clinical information included tumor features and severity such as tumor size, lymph node metastasis and estrogen receptor (ER). We followed up 315 patients among this cohort until July 2014 through regular telephone, clinical visits or email. The overall survival (OS) time was calculated from the diagnostic date to the date of death or last follow-up date.

### Genomic DNA samples

Each participant donated 20 ml of blood into an ethylene diamine tetraacetic acid (EDTA) vacutainer tube used for DNA extraction and genotyping. Total genomic DNA was isolated from whole blood using the QIAGEN DNA Blood Mini Kit (QIAGEN, Inc.). The isolated DNA was stored at −20°C until analysis.

### Genotypes

RFLP-PCR was used to identify the genotypes of the SET8 (rs16917496 C/T) polymorphism within the 3′- UTR in patients and breast cancer cells. The amplification conditions were: 95°C for 5 min, 35 cycles of 95°C for 45 s, 66°C for 40 s, and 72°C for 30 s, and a final extension step of 72°C for 10 min. The primers on the *miR-502* binding site were 5′-GGCCTCACGACGGTGCTAC-3′ and 5′-GTTCCCCAGGAGGATGCT-TAC-3′. A 308-bp DNA fragment was produced in this process, then digested the DNA produced by SWAI (New England BioLabs, Inc.) overnight at 25°C. Allel C lacks the SWAI restriction site and only produces a 308-bp band, allel T produces two bands including 149-bp and 159-bp, TC heterozygote produces three bands (149-bp, 159-bp, and 308-bp). The detailed method of RFLP PCR was described in our previous study [22].

### RNA extraction and quantitative real-time PCR

Total RNA was extracted using Trizol reagents (Invitrogen, USA), according to the manufacturer's instructions. Reverse transcription was performed using M-MLV Reverse Transcriptase (Applied Biosystems, USA). QRT-PCR was performed using ABI 7900 Real-time PCR (Applied Biosystems, USA). Probe for SET8 was 5′-FAM-CCCTGTCCGAAGGAGCTCCAGGAAGA-TAMRA-3′. Forward and reverse primers for SET8 are 5′-CGCAAACTTA CGGATTTCT-3′ and 5′-CGATGAGGTCAATCTTCATT-3′, respectively. Samples were done in triplicate. GADPH were used as an endogenous control to normalize the level of SET8 expression. The relative expression of SET8 was calculated using the 2^−△△ct^ method. SDS 2.4 Software (Appliec BioSystems, USA) was used to analyze data. The detailed protocol was described in our previous study [22].

### Western blot

Protein was extracted from breast cancer tissues and cells at 48 h after transfection with siSET8 using RIPA buffer (Thermo, USA). Protein concentration was measured using the BCA protein quantitation kit by Protein Assay Kit (Bio-Red). Total 40ug of protein mixed with 5× loading buffer was separated by SDS-PAGE electrophoresis and transferred to PVDF membranes. Membranes were blocked with 5% non-fat milk for 1 h, and incubated with diluted 1:2000 SET8 monoclonal antibodies (Thermo, USA) at 4°C overnight. Secondary antibodies were added at concentrations of 1:4000. β-actin was used as an internal reference (Santa Cruz Biotechnology, USA). C-DiGit Chemiluminescent Western Blot Scanner (LI-COR, USA) was used to visualize the protein bands.

### Immunohistochemical (IHC) Staining

Formalin-fixed and paraffin-embedded blocks the breast cancer specimens and were analyzed using anti-SET8 antibody (Abgent, China). The deparaffinized sections were boiled in a sodium citrate buffer (pH 6.0) for 15min as an antigen retrieval method. After quenching endogenous peroxidases with hydrogen peroxide, the sections were rinsed with Tris-HCl buffer twice and incubated with anti-SET8 antibody diluted at 1:800 overnight at 4°C. The primary antibodies were detected using a secondary antibody with HRP polymer (EnVision™ Detection Systems, DAKO, Carpinteria, CA). Diaminobenzidine was used as chromogen according to the manufacture's instruction. Hematoxylin was used for nuclear counterstaining, and the sections were mounted and coverslipped. The tissue staining results were scored based on signal distribution (distribution score) and intensity (intensity score). The distribution score includes 0 (0–5%), 1 (6–25%), 2 (26–50%), 3 (51–75%) and 4 (76–100%), which indicates the percentage of positive cells in all the tumor cells present in a sample. The signal intensity consists of 0 (no signal), 1 (weak), 2 (moderate), or 3 (strong). The final staining score was the product of the distribution and intensity scores.

### TCGA mRNA dataset analysis

SET8 relative expression data of breast cancer patients were obtained from the Cancer Genome Atlas (TCGA) data portal (tcga-data.nci.nih.gov/tcga/). The relative expression data of SET8 consists of normalized (level 3) from the Illumina HiSeq_RNASeqV2 platform and Agilent4502A_07 platform (paired tumor tissue and adjacent normal tissues, *n* = 166). Level 3 normalized mRNA expression was normalized with the quantile method using the R/Bioconductor package of preprocessCore [41].

### Bioinformatic miRNA target prediction and analysis

miRWalk 2.0 database was used to predict potential target genes for miR-502, which combines the information with a comparison of binding sites resulting from other three existing miRNA-target prediction programs: Targetscan6.2, miRNAMap, miRanda-rel2010 [42]. The Venn diagram was drawn by Venny2.1 software. The minimum free energy (MFE) hybridization of miR-502 with C or T genotype of SET8 3′-UTR was predicted using RNAHYBRID software (http://bibiserv.techfak.uni-bielefeld.de/rnahybrid/submission.html) [43].

### Statistical analysis

The expression of SET8 was calculated using the 2^−△△ct^ method, and the variance of SET8 relative expression and the expression of breast cancer marker in different genotypes was calculated using one-way ANOVA method. A chi-squared test was used to show the association between SNP rs16917496 genotypes and clinicopathological features of patients. The differences in survival were examined using the log-rank test, and Kaplan-Meier curve and Cox regression were used to analyze the relationship between genotype and survival time of breast cancer. Statistical analyses were performed with SPSS 19.0 (SPSS Inc., Chicago, IL, USA), and graphs were generated by Graphpad Prism 5.0statistic software (Graphpad Software, Inc.) and STATA 12.0 (version 12.0; StataCorp, College Station, Texas, USA). All statistical tests were two-sided, and *P* value less than 0.05 was considered statistically significant.

## SUPPLEMENTARY MATERIAL FIGURES AND TABLES


