# Supplementary material for: A functional single nucleotide polymorphism of SET8 is prognostic for breast cancer
Source: Oncotarget. 2016 Apr 29;7(23):34277–87. doi: 10.18632/oncotarget.9099 (PMC5085155; doi:10.18632/oncotarget.9099)
Supplement: Supplementary file 1 [file oncotarget-07-34277-s001.pdf]

A functional single nucleotide polymorphism of SET8 is prognostic for breast cancer

Supplementary Materials

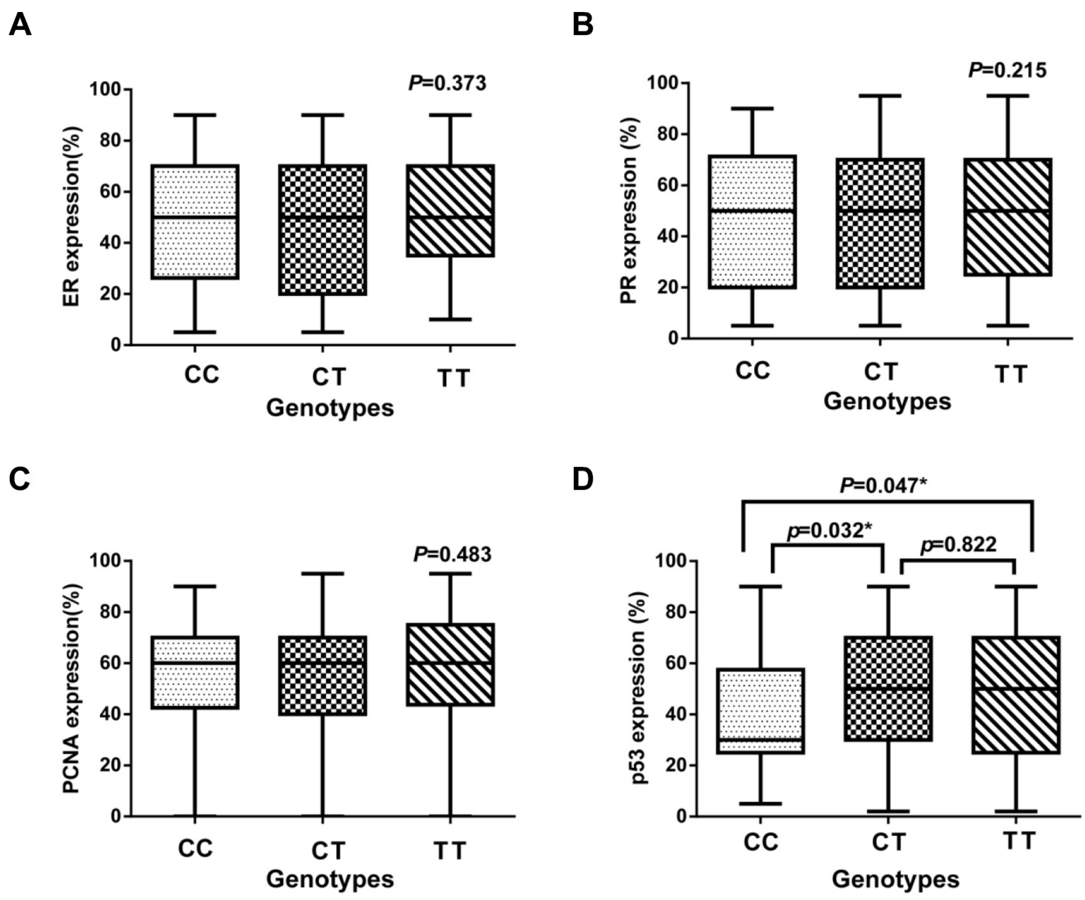

Supplementary Figure S1: Association between SET8 genotype and known breast cancer prognostic parameters. (**A–D**) Association between ER (A), PR (B), PCNA (C) and p53 (D) expression and different rs16917496 genotypes.

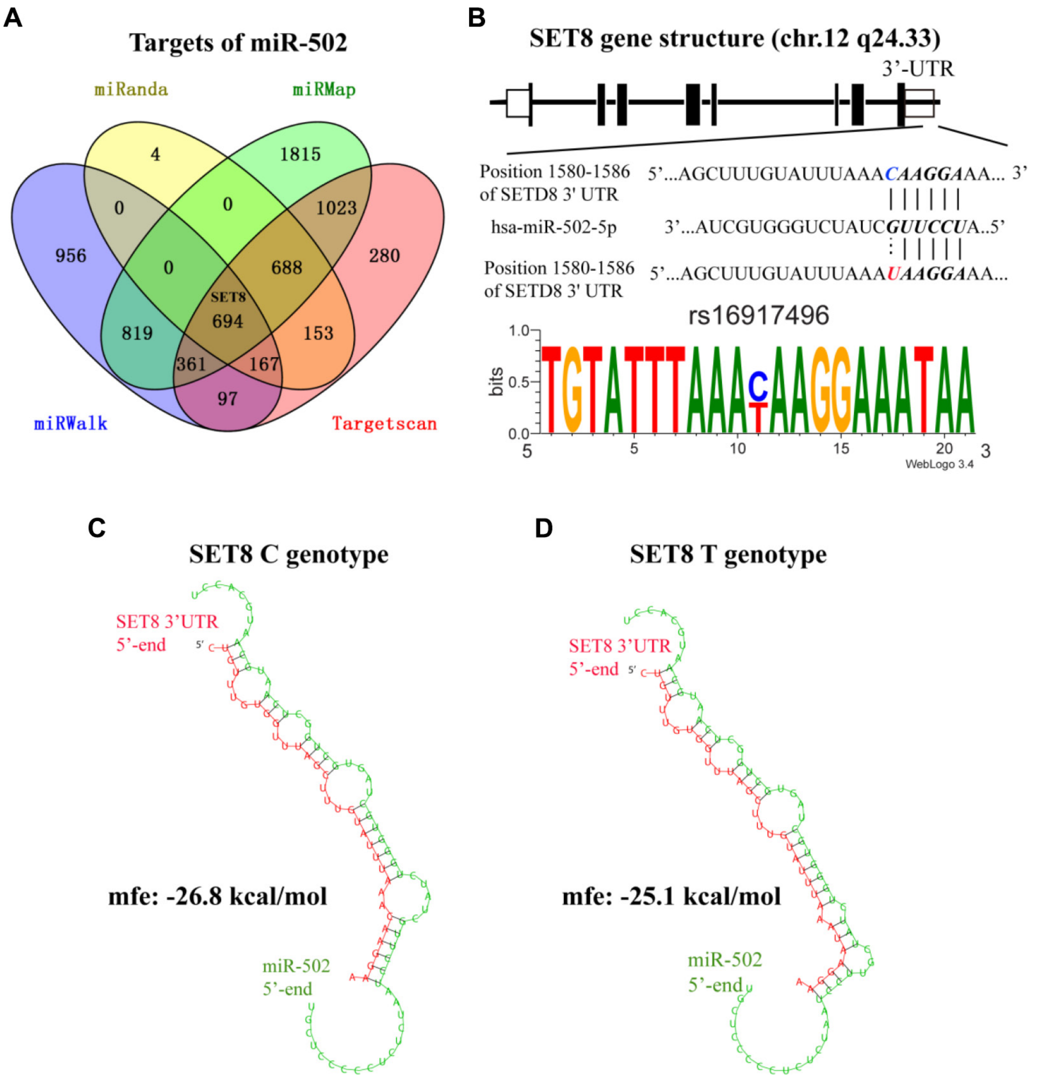

Supplementary Figure S2: Functional prediction of the miR-502 binding site in the SET8 3′-UTR and the effect of SNP rs16917496 on binding affinity. (**A**) A Venn diagram shows the overlap of 4 miRNA target prediction database which identified 694 candidate genes which may interact with miR-502. (**B**) SET8 gene structure and T/C polymorphism in the SET8 3′-UTR at the miR-502 binding site are shown. The altered allele T/C is highlighted. (**C** and **D**) Computational Thermodynamics Modeling of the interaction between miR-502 and SET8 3′- UTR that contains two genotypes was performed on RNAHYBRID software online. miR-502-binding energy for the different alleles in the SET8 3′-UTR is shown.

Supplementary Tables S1: multivariate analysis of overall survival and disease free survival in 315 patients with breast cancer

Factors	Overall Survival		Disease Free Survival	
	HR 95%(CI)	*P*-value	HR 95%(CI)	*P*-value
**Age at diagnosis (≥ 50 vs. < 50)**	3.399 (0.718–16.103)	0.123	5.481 (0.677–44.363)	0.111
**TMN stage (III vs. I + II)**	3.300 (0.647–16.829)	0.151	3.747 (0.761–18.461)	0.104
**Lymph node metastasis (Yes vs. No)**	1.730 (0.300–9.975)	0.540	1.612 (0.302–8.609)	0.576
**Molecular subtype**				
HER-2 overexpression vs. Luminal type	0.675 (0.068–6.713)	0.738	0.539 (0.056–5.174)	0.593
Basal-like vs. Luminal type	5.197 (**1.348–20.027**)	***0.017***	3.870 (**1.273–11.766**)	**0.017**
**Genotype (TT vs.CC+CT)**	3.605 (**1.052–12.352**)	***0.041***	2.564 (0.964–7.615)	0.090
